# Bio-Inspired Propulsion: Towards Understanding the Role of Pectoral Fin Kinematics in Manta-like Swimming

**DOI:** 10.3390/biomimetics7020045

**Published:** 2022-04-15

**Authors:** Alec Menzer, Yuchen Gong, Frank E. Fish, Haibo Dong

**Affiliations:** 1 Department of Mechanical and Aerospace Engineering, University of Virginia, Charlottesville, VA 22903, USA; yg6nc@virginia.edu (Y.G.); hd6q@virginia.edu (H.D.); 2 Department of Biology, West Chester University, West Chester, PA 19393, USA; ffish@wcupa.edu

**Keywords:** high-fidelity flow simulation, bio-inspired locomotion, batoid-like swimming, manta ray

## Abstract

Through computational fluid dynamics (CFD) simulations of a model manta ray body, the hydrodynamic role of manta-like bioinspired flapping is investigated. The manta ray model motion is reconstructed from synchronized high-resolution videos of manta ray swimming. Rotation angles of the model skeletal joints are altered to scale the pitching and bending, resulting in eight models with different pectoral fin pitching and bending ratios. Simulations are performed using an in-house developed immersed boundary method-based numerical solver. Pectoral fin pitching ratio (PR) is found to have significant implications in the thrust and efficiency of the manta model. This occurs due to more optimal vortex formation and shedding caused by the lower pitching ratio. Leading edge vortexes (LEVs) formed on the bottom of the fin, a characteristic of the higher PR cases, produced parasitic low pressure that hinders thrust force. Lowering the PR reduces the influence of this vortex while another LEV that forms on the top surface of the fin strengthens it. A moderately high bending ratio (BR) can slightly reduce power consumption. Finally, by combining a moderately high BR = 0.83 with PR = 0.67, further performance improvements can be made. This enhanced understanding of manta-inspired propulsive mechanics fills a gap in our understanding of the manta-like mobuliform locomotion. This motivates a new generation of manta-inspired robots that can mimic the high speed and efficiency of their biological counterpart.

## 1. Introduction

Researchers have taken an interest in studying the methods by which aquatic animals are able to efficiently swim with the goal of understanding the hydrodynamics principles that nature uses to its advantage to influence swimming performance. Knowledge of these hydrodynamic principles has been utilized in the design of bio-inspired robots to achieve efficient lift and thrust production [1,2,3]. For swimming locomotion, two thrust production methods are utilized by animals. The added-mass effect is one such method in which the swimmer accelerates a mass of water posteriorly near the body using an undulatory motion inducing an equal but opposite thrust force [4]. Leading edge vortex (LEV)-based thrust is the other mechanism. LEV formation from the lateral motions of the caudal fin of a Jackfish-like model is shown to strengthen with the addition of body and anal/dorsal fin interactions. This improves caudal fin thrust generation via a caudal fin capture phenomena [5]. Similar results are observed for a sunfish-like model with carrangiform motion [6]. In addition, sharp and elongated caudal fin geometries have been demonstrated to promote LEV attachment to the caudal fin of a reduced-order fish model [7].

The swimming motions of batoid fishes (e.g., rays and skates) differ greatly from the previously mentioned fish locomotion types. Firstly, batoid fishes are characterized by their dorsoventrally flattened profiles and use of their large pectoral fins for generating thrust [8]. Rosenberger sub-divides batoid locomotive types using a continuum ranging from posteriorly directed undulations of the pectoral fin, or rajiform locomotion, to dorsoventral oscillations in a bird-like flapping motion, or mobuliform locomotion. [9,10]. The literature surrounding hydrodynamic mechanisms of batoid swimming is far less robust than the body and tail undulations of other swimming species described earlier.

The existence of LEV-based thrust production in oscillatory batoid swimming was observed experimentally by Moored et al. [11]. The numerical investigation of batoid rays, including manta and stingrays revealed that enhanced LEV attachment to the propulsive pectoral fins led to improved thrust performance at fast swimming speeds while the added-mass effect dominated thrust production at lower speeds. It should be noted that stingrays fall into the undulating batoid locomotion category [12]. A cownose ray model exhibiting oscillatory motion (wave numbers ranging from 0.0 to 0.8) in the mobuliform swimming mode has been documented to produce LEV induced suction force contributing to thrust. Motion with small wavenumber chordwise deformations, resulting in lower fin pitching angles, increased force production due to stronger LEVs. Despite this, less of the resulting hydrodynamic force could be directed in the streamwise direction [13]. Cownose ray pectoral fins can adopt undulatory or oscillatory behavior [14]. Other studies characterized the flow field and features produced by batoid-inspired models prescribed with batoid-like motion [15,16]. 

Investigation of the role of pectoral fin kinematics in manta-like (mobuliform) swimming does not exist despite the observation and characterization of manta ray swimming [17] and even maneuvering [18]. Detailed observations of manta pectoral fins highlight the presence of both spanwise and chordwise flexibility with extreme chordwise flexibility present near the fin tips. The spanwise and chordwise motions of the fin are markedly asymmetric between the upstroke and the downstroke [17], a feature that has had minimal prevalence in previous studies, although the indication is that the downstroke generates more thrust than the upstroke [17,19]. Manta ray inspired models and kinematics are of interest due to findings that suggest that mobuliform would be an ideal platform for designing a fast swimming, efficient, and maneuverable robotic system [19]. The maximum efficiency of mobuliform swimming has been estimated to be about 0.89, which is higher than the estimates for rajiform swimmers [17,20].

Despite the lack of manta-like geometry and kinematics research, much research has been conducted to understand the hydrodynamic performance and produced wake structures for bio-inspired flapping and pitching-heaving foils. Twist morphing, which introduces spatial variation in pitching angles along the span of a plate, is shown to increase efficiency by 26% due to minimal losses in thrust while significantly reducing power consumption. Increased twisting also concentrates flow bifurcation downstream from the plate, which further enhances efficiency [21]. Additionally, better lift production and lift economy can be achieved with twist-like deformations for a large aspect ratio flapping wing [22]. Decreased pitching angle for a 2-D pitching heaving flat plate, which leads to higher effective angles of attack, have also been shown to lead to stronger LEV structures but with earlier vortex separation [23]. 

Spanwise wave deformations with high amplitudes can strengthen the LEV, thereby increasing the thrust production of a pitching heaving plate by 20%, which is reflected by an increase in mean flow velocity in the downstream wake. Maximal efficiency is reached at small amounts of spanwise bending due to the associated power consumption increase [24,25]. In addition, the combination of chordwise and spanwise deformation waves on an elliptic plate resulted in minimal thrust sacrifice and high power consumption [26]. As mantas exhibit flexible fin deformations, the promising findings regarding performance enhancements for deforming flapping wings prompts research on the role of pectoral fin deformations in manta-like swimming.

The goal of this work is to extend our knowledge of batoid swimming to manta-like geometry and kinematics, thereby building upon previous works that have established an understanding of the characteristics of both bio-inspired flapping models and other more simplistic batoid-like models. This will enable a better understanding of how to leverage manta-like kinematics for propulsive robots. To achieve this, a 3-D manta ray model to which manta-like pectoral fin motion derived from true biological motion is applied. CFD simulations are performed to resolve the performance of the models. In Section 2, the model kinematics and numerical methods are outlined. In Section 3, the results of the numerical simulations are presented and discussed. Concluding remarks are made in Section 4. 

## 2. Materials and Methods

### 2.1. 3D Manta Ray Model Kinematics

The current study employs a manta ray model with motion reconstructed from videos of manta ray (*Mobula birostris*) steady swimming [17] using the 3D modeling software Autodesk^®^ Maya. A joint-based skeletal structure bound to a polygonal-mesh skin for modeling the manta motion is used in this study. This technique has been successfully applied to reconstructions of bird flight and tuna body with caudal fin and finlet motion [27,28]. The manta ray model includes complex geometric and kinematic features of its biological counterpart, producing a 3-D model with manta-like swimming motion. This was achieved by rotating the skeletal joints so that the model manta pectoral fin closely matched that of the manta ray captured on video. Joint rotation values were then fitted to a Fourier series allowing prescribed manta-like motion to be applied to the model. Prescribing motion in this manner allows for ease of kinematics scaling, as will be explained later. 

**Figure 1:**
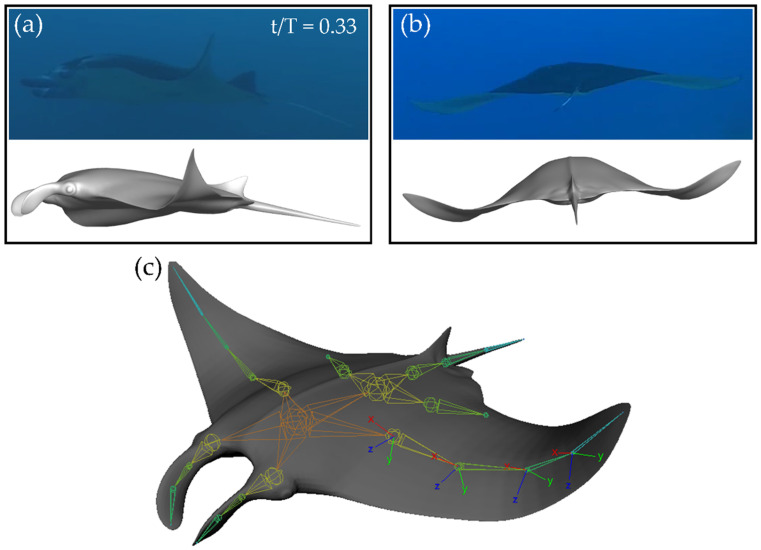
Comparison between biological motion and prescribed manta-like motion from side (**a**) and back (**b**) views at T = 0.33. (**c**) Skeletal joint arrangement for the current model.

As shown in Figure 1a, the model viewed from an approximately side-on angle matches closely to that of the biological manta ray during the downstroke. Viewed along the z-axis in Figure 1b, close matching of the motion is also observed. The organization of the skeletal joints can be seen in Figure 1c. Local coordinate axes for each joint are shown. Local x-axes point parallel to the span, local y-axes point perpendicular to the span out of the fin, and local z-axes point perpendicular to the span along the chord of the fin.

**Figure 2:**
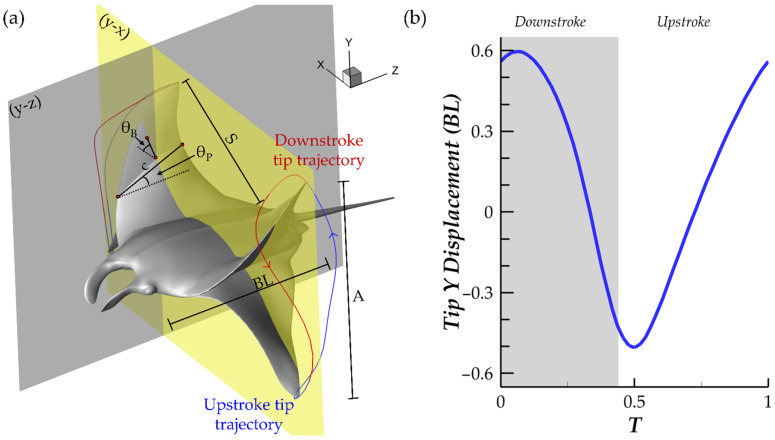
(**a**) Geometric variables for the manta ray model along with fin tip trajectories illustrating manta-like flapping motion during the downstroke (red trajectory) and upstroke (blue trajectory); (**b**) fin tip y-displacement.

A detailed look into the model geometric parameters as well as a visualization of the flapping motion of the model can be found in Figure 2a. The body length (BL), pectoral fin span (*S*), pectoral fin chord (c), tip flapping amplitude (A), pitching angle (*θ_P_*), and bending angle (*θ_B_*) are illustrated. It should be noted that *c* changes with the position along *S. θ_B_* is defined at the mid-chord along the span of the fin. *θ_P_* represents the orientation of a chord with respect to the z-axis in the y-z plane. *θ_B_* represents the orientation of the fin at the mid-chord with respect to the x-axis in the y-x plane. The fin tip trajectories are also displayed for the downstroke (red) and upstroke (blue). During stroke reversal, the tip motion lags behind the motion of the rest of the fin, which is reflected by stroke reversals occurring before the minimum and maximum fin tip positions are achieved. This agrees with observations of manta ray pectoral fin motion during forward swimming [17,19]. Figure 2b displays the y-displacement of the fin tip during a full cycle of flapping. The observed pattern in which the tip vertical position undergoes a smooth ‘semi-circular’ trajectory above the body mid-plane and a sharper ‘triangular’ trajectory below the body mid-plane agrees with biologist observations of the manta ray pectoral forward swimming fin tip trajectory [17].

**Figure 3:**
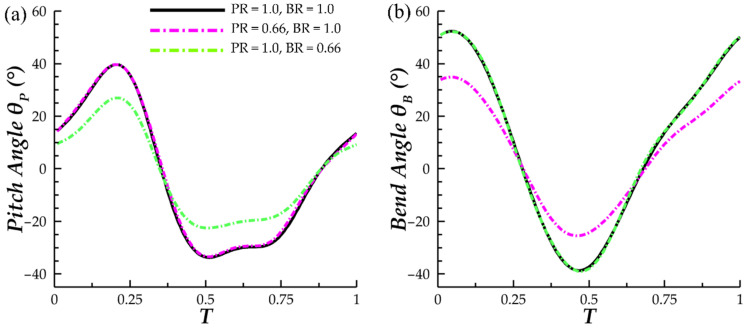
Comparison of (**a**) *θ_P_* and (**b**) *θ_B_* at pectoral fin span *s/S = 0.5* for three PR and BR combinations.

To obtain models with modified motion, the planar angles of the pectoral fin orientation, *θ_P_* and *θ_B_*, were scaled independently. This was achieved through scaling of the skeletal joint local x-rotation and z-rotation angles that are prescribed to the pectoral fin joints. These joint rotation angle scaling ratios are denoted as *rx* and *rz*, respectively. As the joint local coordinate axes and global coordinate axes are not incident, a mapping between *(rx, rz*) and (*θ_P_*, *θ_B_*) is necessary in order to obtain the correct *rx* and *rz* for a desired (*θ_P_*_,_ *θ_B_*) combination. Using MATLAB, mapping and solving for the correct *rx* and *rz* was performed at each key time frame of the model. This information was stored in a format that is readable by Maya and could then be applied to the joints. This produced flapping motion with independent scaling of pectoral fin bending and pitching angles. In Figure 3a,b, the pectoral fin *θ_P_* and *θ_B_* plots at *s/S = 0.5* can be found for cases with varying pitch ratio (PR) and bend ratio (BR), where PR denotes *θ_P,scaled_/θ_P,unscaled_* and BR denotes *θ_B,scaled_/θ_B,unscaled_*. The independence of *θ_P_* and *θ_B_* is clear. 

For this study, 8 cases are constructed. The first is the baseline case with unscaled motion. Cases 1–3 represent fixed BR and decreasing PR. Cases 4–6 represent fixed PR and decreasing BR. Finally, case 7 is simultaneous scaling of BR and PR. For ease of reference, Table 1 provides a summary of the cases in tabular form.

### 2.2. Numerical Methods and Simulation Setup

The governing fluids equations solved in this work are the incompressible Navier–Stokes equations. They are displayed in indicial form in Equation (1) where *u_i_* are the velocity components, *p* is the pressure component, and *Re* is the Reynolds number.

(1) $$\frac{\partial u_{i}}{\partial x_{i}}=0;\;\;\frac{\partial u_{i}}{\partial t}+\frac{\partial u_{i}u_{j}}{\partial x_{j}}=-\frac{\partial p}{\partial x_{i}}+\frac{1}{Re}\frac{\partial^{2}u_{i}}{\partial x_{i}\partial x_{j}}\;$$

To solve these equations an in-house finite-difference based Cartesian-grid sharp-interface immersed-boundary method direct numerical simulation solver (DNS) is employed. The DNS used in this study employed a second order center scheme for spatial discretization and a second order fractional time step method for temporal discretization. Convective terms in (1) are discretized using the Adams–Bashforth scheme and diffusion terms in (1) are discretized using an implicit Crank–Nicolson scheme. A fast multi-grid method (MG method) is used to solve the pressure Poisson equation. The pressure convergence stopping criteria is set to 10^−3^ in all the cases. In depth information regarding this solver can be found in Ref. [29]. Validations for the numerical solver can be found in Refs. [27,30,31]. Recent applications of the solver used in the current study for biologically-inspired swimming used in the current study include inline flapping foils, flapping foil geometry optimization, dense fish schools, and Crevalle jackfish swimming [32,33,34,35]. CFD is a powerful tool for studying fluid-related biological phenomena from microorganisms to larger flying and swimming animals (as with the current study). Finite volume methods have been employed to study microorganisms over a range of applications from mixing in bioreactors to airborne microorganism spread [36,37]. The current study implements the finite difference method for its capability to resolve a wide range of fluid motion scales as well as its suitability for handling the complex manta model geometry and body motion.

**Figure 4:**
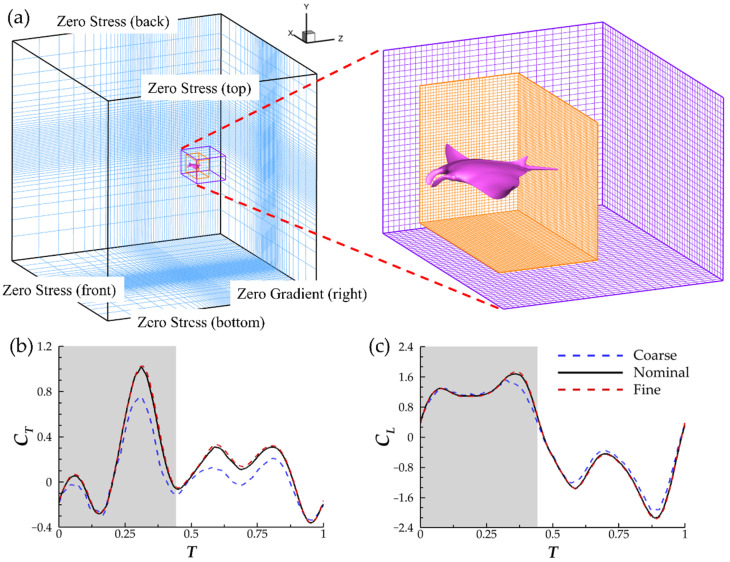
(**a**) Schematic of the computational domain and boundary conditions used in the present study. (**b**) Instantaneous thrust and (**c**) lift comparison between the coarse mesh (∆ = 0.018 BL, ~4.3 million nodes) nominal mesh (∆ = 0.0085 BL, ~8.7 million nodes), and fine mesh (∆ = 0.0065 BL, ~15.4 million nodes).

The computational domain has dimensions of 20 BL × 20 BL × 20 BL. A Cartesian grid configuration with a stretching grid is used. To achieve suitable density in the near-field regions without utilizing a dense base mesh, adaptive mesh refinement (AMR) techniques are utilized. Two block-structured-mesh bodies are implemented. A large parent block captures far-field wake structures downstream of the model while a smaller block with boundaries closer the model enhances grid density in the near-field. For maximal efficiency of the AMR algorithm, the incoming flow velocity is set to be in the z-direction. The left boundary of the domain is set to be the velocity inlet. The computational domain, including the positions of AMR blocks, can be found in Figure 4a. Every 4th grid point is shown in the dense region and each AMR block so that meshes are visible. More information on the algorithm can be found in Ref. [38]. 

The spacing of the cells in all 3 spatial directions was determined through a grid independence study. The time increment for each simulation is set as ∆t = 1/960. Peak |*C_T_*| for coarse grid density and fine grid density vary from the nominal grid density by 24% and 1.1%, respectively. Meanwhile, peak |*C_L_*| differs from the nominal density by 8.9% for the coarse grid density and 1.2% for the fine grid density. This demonstrates sufficient accuracy of the nominal grid density for the simulations. Further comparisons between the instantaneous thrust and lift coefficients can be found in Figure 4b,c. The grid study was conducted at *Re* = 1200. Manta rays typically operate in higher Reynolds number regimes between 6.85 × 10^5^ and 7.71 × 10^6^ [17]. However, performing DNS on flows with Reynolds numbers, this high value is prohibitively computationally expensive. Inertial forces still dominate the flow at *Re* = 1200 and thus it is used for this study. 

To quantify the hydrodynamic performance of each model, thrust is defined as the pressure force opposite of the z-direction, drag is defined as shear force in the z-direction, and lift force as pressure force in the y-direction. Force/power coefficients and efficiency are defined as:

(2) $$C_{T,D,L}=\frac{\mathrm{Thrust},\mathrm{Drag},\mathrm{Lift}}{\frac{1}{2}{\rho U}_{\infty }{}^{2}\mathrm{BL}},{\;C}_{\mathrm{PW}}=\frac{\mathrm{Power}}{\frac{1}{2}{\rho U}_{\infty }{}^{3}\mathrm{BL}},\;\eta =\frac{\bar{C_{T}}}{\bar{C_{PW}}}$$

(3) $$Re=\frac{U_{\infty }\mathrm{BL}}{\nu };\;\;St=\frac{A\;f}{U_{\infty }}$$

where $\bar{C_{T}}$ and $\bar{C_{PW}}$ represent the thrust production and power consumption averaged over a cycle of motion. Non dimensional flow parameters Strouhal number, *St*, and Reynolds number, *Re,* are shown in Equation (3). ν represents kinematic viscosity.

To obtain the flow parameter *St* for the current study, the free stream velocity $U_{\infty }$ that produced free swimming (i.e., cycle averaged force and drag balance) for the baseline case (with PR = 1.0 and BR = 1.0) was found. This velocity in its nondimensional form is $U_{\infty }\;$*=* 1.25 BL/cycle. The baseline case manta model flaps at a frequency f of 1.0 s^−1^ and a fin-tip amplitude A of 1.10 BL. Thus, the effective fin tip Strouhal number, *St_tip_*, for all cases is set at 0.88. This high *St* at the fin tips is expected to lead to enhanced thrust production in the distal regions of the fin [39] and is similar to the effective tip *St* of models used by Fish et al. [17], Zhang et al. [13], and Liu et al. [39]. 

**Figure 5:**
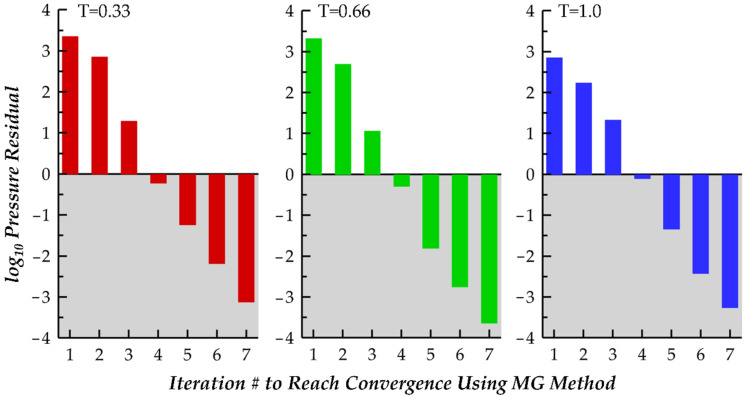
Illustration of Poisson equation pressure convergence for 3 time steps during a cycle of motion: T = 0.33 (320th time step), T = 0.66 (640th time step), and T = 1.0 (960th time step).

Simulations were performed on the University of Virginia’s high performance computing cluster. Each time step took approximately 7.5 CPU core minutes to evaluate, and plots of the pressure residuals for three time steps, T = 0.33, T = 0.66, and T = 1.0, are shown in Figure 5. Further, each simulation used 9 CPU cores, and the simulation of 1 cycle of motion required approximately 120 core hours to complete. 

## 3. Results and Discussions

In this section, the hydrodynamic performance and produced wake structures are examined for the cases described in Section 2.1. To ensure that force production reached steady periodicity, each of the models was simulated for 4 cycles of motion. Compared to the 3rd cycle of motion, the peak thrust achieved in the 4th cycle of motion differs by less than 1%. Cycle averaged thrust values from the 4th cycle of motion are also within only 0.5% of the 3rd cycle of motion. We conclude that periodic force production has been reached at the 4th cycle of motion. Firstly, hydrodynamic performance will be examined. 

### 3.1. Comparison of Hydrodynamic Performance for Varying Bending and Pitching

**Figure 6:**
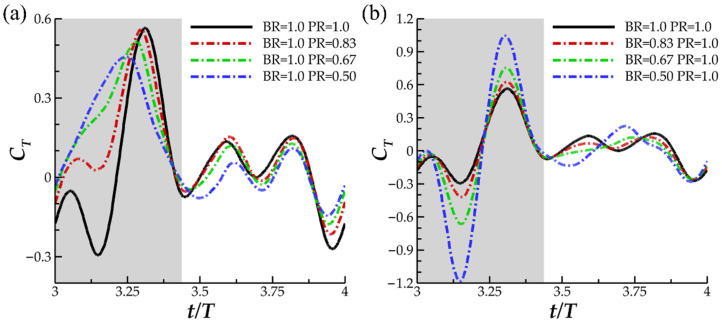
Instantaneous C_T_ for (**a**) the baseline case (BR = 1.0 PR = 1.0), case 1 (BR = 1.0 PR = 0.83), case 2 (BR = 1.0 PR = 0.67) and case 3 (BR = 1.0 PR = 0.50); (**b**) the baseline case (BR = 1.0 PR = 1.0), case 4 (BR = 0.80 PR = 1.0), case 2 (BR = 0.67 PR = 1.0) and case 3 (BR = 0.50 PR = 1.0).

In Figure 6, the instantaneous thrust for varying PR (a) and varying BR (b) are displayed. In Figure 6a, noticeable differences between thrust production are observed in the downstroke phase of the flapping motion. For the baseline case, two thrust peaks, one occurring early in the downstroke and the other late in the downstroke, are separated by a trough occurring at t/T = 3.15. Reverse thrust is produced for a significant portion of the downstroke until t/T = 3.21. A similar trend of two thrust peaks occurs for case 2 (with PR = 0.83). However, reverse thrust is not produced. For cases 2 and 3, representing lower PR, gradual ascension to peak thrust is exhibited, and peak thrust occurs earlier in the downstroke motion. During the upstroke motion, thrust production is consistent across each of the cases and is characterized by a double peak shape.

In Figure 6b, the peak-valley-peak pattern of downstroke thrust production observed for the baseline case is emphasized with decreasing BR. The thrust valley for case 6, with BR = 0.50, produced a higher magnitude reverse thrust (|C_T_| = 1.18) than peak downstroke forward thrust (|C_T_| = 1.04). As for the upstroke, the double peak in thrust production for the BR = 1.0 PR = 1.0 case transitions to a single peak for BR = 0.5. The observed upstroke-downstroke asymmetry in force production is a product of the temporally asymmetric manta-like motion of the pectoral fin. The downstroke, which lasts until T = 0.43, is quicker than the upstroke which contributes to the increased thrust production during this phase. In addition, the temporal pitching and bending asymmetry, shown in Figure 3a,b, contributes as well. The highest pitching and bending angles are achieved during the downstroke portion of the motion, which contributes to biased force production. Such extreme asymmetric motion applied to a batoid model has not yet been researched, and as such, the force patterns produced by the current models is not similar to any previous studies. 

Cycle averaged $\bar{C_{T}}$, $\bar{C_{PW}}$ and efficiency η can be found in Table 2. The table entries are normalized by the baseline case performance for which $\bar{C_{T}}$ = 0.040, $\bar{C_{PW}}$ = 0.728, and η = 0.054. In the row below normalized $\bar{C_{T}}$, the decomposition of the thrust into contributions by the upstroke and downstroke, displayed as fractions of the cycle thrust production, is shown for further comparison. 

Clear trends in power consumption and thrust are seen. Cases 1–3 represent a decrease in PR from left to right while cases 4–6 represent a decrease in BR from left to right. Dramatic thrust improvements are observed for cases 1–3. Accompanied by only a slight increase in power consumption compared to the baseline case, case 2 exhibits optimal efficiency. Thrust decreases for cases 4–6 while power consumption reaches a minimum for case 4, where BR = 0.83. In comparison to the baseline case, though, efficiency is not improved due to lower thrust values. 

Large differences between downstroke and upstroke thrust production are noticed in Figure 5 and the thrust decomposition into % contributions are shown in Table 2. Decreasing PR for cases 1–3 leads to an increase in the downstroke thrust contribution, which corresponds to a decrease in upstroke thrust contribution. Case 2, with maximum $\bar{C_{T}}$, interestingly demonstrates nearly all thrust production during the downstroke. In case 3, where PR is lower than case 2, the downstroke contributes more thrust than is produced on the cycle average but at the cost of reverse thrust during the downstroke. 

Zhang et al. [13] reported an increase in power consumption as chordwise deformations are reduced and there is an increase in maximal thrust for moderate chordwise deformations. It should be noted that increased chordwise deformations led to a decreased fin effective angle of attack. In the current study, an increasing PR leads to a decreasing angle of attack. Thus, the observed balance between increased power consumption and thrust production in the current study agrees with Zhang et al. [13]. Previous work investigating spanwise deformations on flapping wings report an increase in thrust as spanwise bending deformation amplitude increases. In addition, efficiency reaches a peak for smaller amplitude deformations [24]. Similar trends are observed for the current models for increasing thrust as spanwise bending angles increase. However, discrepancies arise for efficiency. This difference is reconciled by a study on cownose ray pectoral fin performance where both efficiency and thrust are reported to increase with higher bending angles [40]. The geometry and kinematics of the cownose ray model more closely match those used in the current study. 

The trend of slight power consumption reduction for moderately high BR and significant thrust increase for moderate PR suggests the combined effect of lowering BR to 0.83 and PR to 0.67 that may produce further performance enhancements. This will be investigated in Section 3.4.

### 3.2. Comparison of Vortex Topology

**Figure 7:**
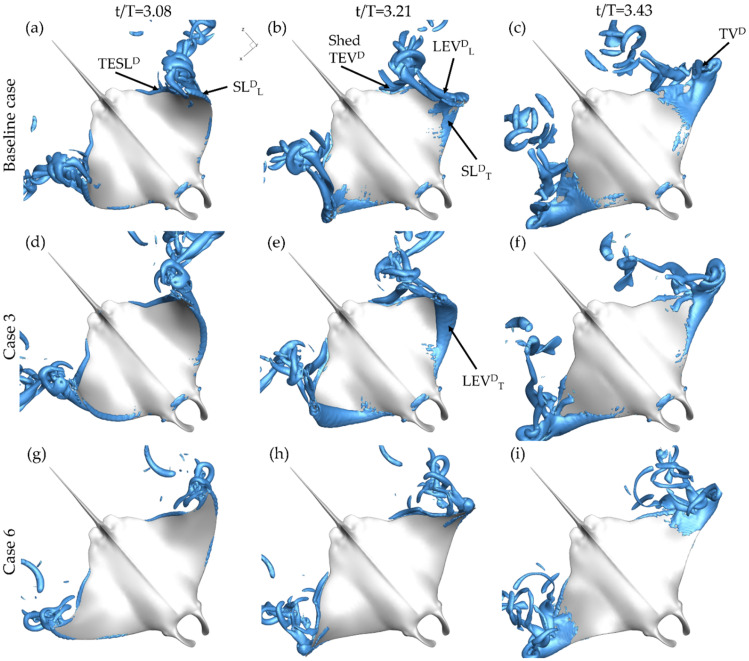
Perspective views of downstroke vortex formation for the baseline case (BR = 1.0 PR = 1.0) (**a**–**c**) case 3 (BR = 1.0 PR = 0.50) (**d**–**f**) and case 6 (BR = 0.5 PR = 1.0) (**g**–**i**) at t/T = 3.08 (**a**,**d**,**g**) t/T = 3.21 (**b**,**e**,**h**) and t/T = 3.43 (**c**,**f**,**i**).

In this section, vortex generation on the pectoral during the downstroke and upstroke will be examined. To illustrate key flow features, iso-surfaces are visualized using a Q-criterion value of 120. First, the downstroke vortex formation is examined. In Figure 7a top-down view of the baseline case (a–c), case 3 with PR = 0.5 (d,f), and case 6 with BR = 0.5 (g–i) cases are provided. Cases 3 and 6 were selected for comparison so that the maximal difference in variation of BR and PR could be examined. The figure is further divided into three times during the downstroke: t/T = 3.08 (a,d,g), t/T = 3.21 (b,e,h), and t/T = 3.43 (c,f,i) in order to capture formation and shedding of structures during the whole downstroke. To reference the structures, the following notation scheme is utilized: vortex type followed by a superscript indicating occurrence during the upstroke (U) or downstroke (D) and a subscript indicating whether the formation occurs on the top (T) or lower (L) surface of the fin.

At t/T = 3.08, key differences in the downstroke leading-edge vortex formation on the top surface of the manta body, LEV^D^_T_, are displayed. No top surface downstroke shear layer (SL^D^_T_) or LEV^D^_T_ is observed for baseline case in Figure 7a or for case 6 in Figure 7g. Both the baseline case and case 6 have PR = 1.0. Yet, case 3 with PR = 0.50 in Figure 7d demonstrates LEV^D^_T_ formation on the top of the fin even at the early stage of the downstroke. A notable difference is also observed towards the tip of the of the manta fin. A SL formed on the lower surface of the fin during the early downstroke, denoted SL^D^_L_, is observed near the tip of the fin for the baseline case and case 3 in Figure 7a,g. Progressing to about halfway through the downstroke, case 6 in Figure 7h still exhibits no LEV^D^_T_ formation while the baseline case in Figure 7b has progressed from no formation to clear SL^D^_T_ formation. In addition, the SL^D^_L_ for each of the cases with PR = 1.0 (the baseline case and case 3) has strengthened to form an LEV^D^_L_ as seen in Figure 7b,e. At this time step, for each of the three cases, the trailing-edge shear layer (TESL^D^) has rolled up on the under-surface of the fin to produce the downstroke trailing-edge vortex (TEV^D^) seen separating from the back of the fin in each of Figure 7d–f. Finally, at the end of the downstroke, top surface vortex separation near the fin tips is observed for all cases. In particular, for the baseline case in Figure 6c and case 3 in Figure 7f, a tip vortex (TV^D^) is seen extending upwards. The TV^D^ is much less pronounced for case 6 case as seen in Figure 7i. In general, the vortexes produced by the baseline case and case 6 appear less robust than those produced by case 3. 

**Figure 8:**
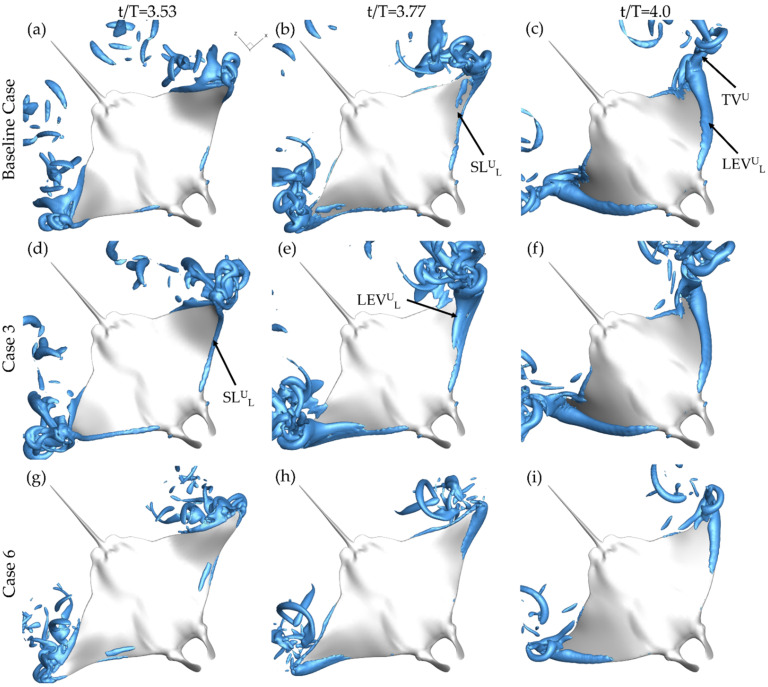
Perspective views of upstroke vortex formation for the baseline case (BR = 1.0 PR = 1.0) (**a**–**c**) case 3 (BR = 1.0 PR = 0.50) (**d**–**f**) and case 6 (BR = 0.5 PR = 1.0) (**g**–**i**) at t/T = 3.53 (**a**,**d**,**g**) t/T = 3.77 (**b**,**e**,**h**) and t/T = 4.0 (**c**,**f**,**i**).

Figure 8 provides a bottom up view of the same cases at t/T = 3.53 (a,d,g), t/T = 3.77 (b,e,h), and t/T = 4.0 (c,f,i). In the beginning of the upstroke, the LEV formation on the lower surface of the fin, indicated by LEV^U^_L_, follows a similar pattern to that of the LEV^D^_T_ formation. Case 3, displayed in Figure 8d, shows a more developed SL^U^_L_ than both the baseline case and case 6 shown in Figure 8a,g, respectively. At the half-upstroke, each of the cases demonstrates SL^U^_L_ formation. However, the size and location differ greatly. As shown in Figure 8b, the shear layer is only minimally present along the fin for the baseline case. For case 6 in Figure 8e, the SL^U^_L_ covers a larger portion of the fin while an LEV^U^_L_ tube, formed during the first half of the upstroke, has already separated from the surface. Figure 8h shows SL^U^_L_ formation occurring only near the distal regions for case 6. Finally, at the end of the downstroke, LEV^U^_L_ tubes can be seen for each of the models as well as TV^U^_L_ structures that extend from the LEV^U^_L_ tubes.

From the above analysis, clear differences arise in vortex formation for the varying PR and BR. The most significant involve the LEV^D^_L_ and LEV^D^_T_ formation. LEV^D^_T_ formation is delayed and the presence of LEV^D^_L_ occurs due to higher PR, meanwhile, LEV^U^_L_ formation is concentrated near the tip of the fin by lower BR. Observed LEV^D^_T_ and LEV^U^_L_ formation is consistent with the findings made by Zhang et al. [13]. The highest chordwise deformation, characterized by lower fin effective angle of attack, demonstrated the least developed LEV structure [13]. For the current study, earlier LEV^D^_T_ development for the higher effective angle of attack, demonstrated by case 3 with PR = 0.50, is consistent with findings for a canonical pitching heaving foil [23]. The LEV^D^_L_ formation for cases with high pitching ratio has not been shown to occur for other models, however, which may be due to the extreme range of motion of pitching and bending exhibited by the current manta models. 

The next section aims to discuss in further depth how the formation of these vortexes on the fin impacts the generated hydrodynamic forces. LEV^D^_T_ and LEV^D^_L_ formation has been shown to be significantly different as PR is changed, which is reflected by the significant change in downstroke thrust production, seen in Figure 6a. This analysis will enhance our understanding of how performance is impacted by the fin kinematics. 

### 3.3. Impact of LEV and AV Formation on Hydrodynamic Surface Pressure

To better understand the LEV^D^_L_ formation, ω_y_, vorticity contours and vorticity magnitude plots are shown. The orientation of the fin near the tip is such that the y-direction closely matches the spanwise direction of the fin. The corresponding impact on surface pressure and hydrodynamic force is visualized through pressure iso-surface and surface pressure contours. 

**Figure 9:**
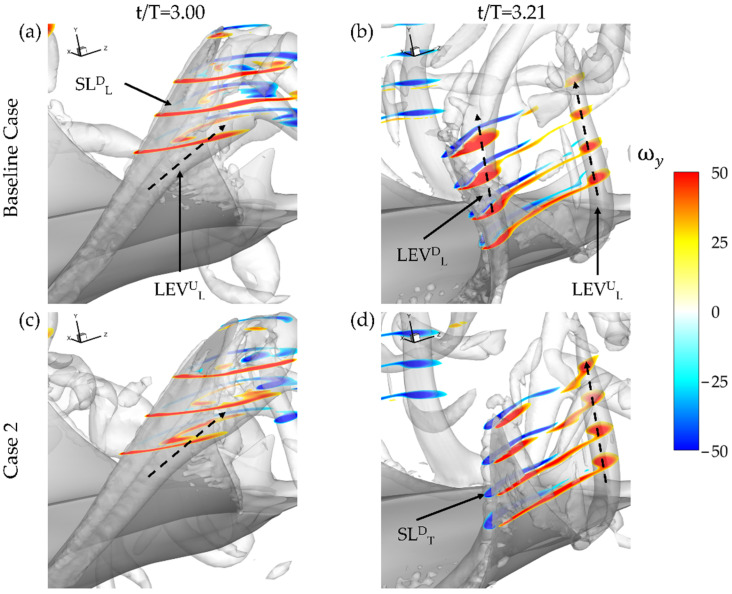
Spanwise vorticity plots for the baseline case (BR = 1.0 PR = 1.0) (**a**,**b**) and case 2 (BR = 1.0 PR = 0.67) (**c**,**d**) at t/T = 3.0 (**a**,**c**) and t/T = 3.21 (**b**,**d**).

As described in Section 3.2 and visualized in Figure 7b, LEV^D^_L_ formation on the undersurface of the fin for the baseline case is present. The highest performing thrust model, case 2 with BR = 1.0 PR = 0.67, does not exhibit the LEV^D^_L_ as seen in the ω_y_ plots shown in Figure 9. Iso-surfaces, visualized by a Q-criterion value of 150, are also included to better illustrate the overall vortex arrangement. At the beginning of the downstroke, the previously formed LEV^U^_L_ can be seen a detached from the fin for both cases in Figure 9a,c. Only slight differences between the relative size of the high vorticity regions of the SL^D^_L_ between the two cases is evident. At the half downstroke, the presence of the LEV^D^_L_ is clear for the baseline case with a tube-like structure extending from the bottom surface in Figure 9b. In contrast case 2 shown in Figure 9d has no such LEV^U^_L_ formation occurring. This is accompanied by SL^D^_T_ formation indicated by the regions of counter-vorticity on the top surface of the fin. The previously shed LEV^U^_L_ has a convected downstream from the model for each of the cases as well.

**Figure 10:**
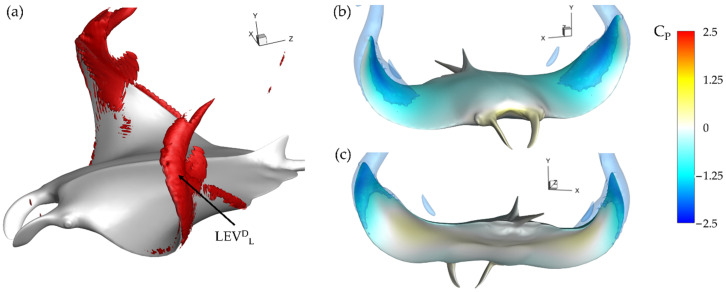
Baseline case (BR = 1.0 PR = 1.0) (**a**) Vorticity magnitude at half downstroke with (**b**,**c**) resulting surface pressure on top and bottom, respectively.

This can be further visualized in Figure 10a through iso-surfaces depicting vorticity magnitude and in Figure 10b,c where the resulting impact on surface pressure at t/T = 3.21 is shown. In Figure 10a iso-surfaces are visualized using a vorticity magnitude value of 90, while in Figure 10b,c iso-surfaces are visualized using a C_P_ value of −1.0. For the baseline case in Figure 10a, the lack of SL^D^_T_ or LEV^D^_T_ development is clear as only a thin layer of vorticity is present on the fin near the trailing edge of the distal region. This contrasts with the lower surface where a strong LEV^D^_L_ is visible. The lower surface LEV^D^_L_ results in a parasitic low-pressure region, which is illustrated in Figure 10c. No such strong thrust producing low pressure region due to vortex formation exists on the top surface as seen in Figure 10b. 

This helps to explain the ‘double-peak’ feature of the instantaneous C_T_ for the BR = 1.0 PR = 1.0 case seen in Figure 6. The reverse thrust produced by the manta model is apparently caused by the attachment of the LEV^D^_L_ on the lower surface, which produces detrimental low pressure on the bottom of the fin. This is compounded by weak LEV^D^_T_ formation as the lack of thrust generating low pressure on the top surface causes the low suction pressure on the bottom of the fin to dominate. The low pressure on the bottom of the fin destructively interferes with any thrust produced by low pressure on the top surface.

**Figure 11:**
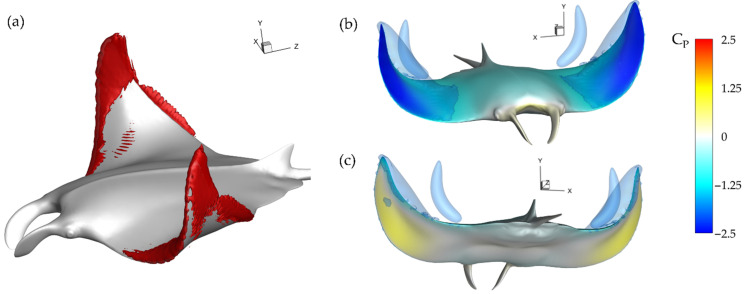
Case 2 (BR = 1.0 PR = 0.67) (**a**) Vorticity magnitude at half downstroke with (**b**,**c**) resulting surface pressure on top and bottom, respectively.

Examining the thrust plot in Figure 6a for the highest $\bar{C_{T}}$ case, case 2, the period of reverse thrust does not occur. Figure 11a illustrates the near body vorticity and Figure 11b,c the surface and iso-surface pressures at t/T = 3.21. Noticeable differences between the vorticity field for the baseline case in Figure 10a and case 2 in Figure 11a are apparent. Namely, regions of high vorticity can be located across most of the leading edge of the fin case while the strong LEV^D^_L_ is not formed at all. As a result, extremely strong low-pressure regions can be observed on the top surface of the fins in Figure 11b while high pressure regions with minimal parasitic low pressure are seen on the bottom surfaces in Figure 11c. Thus, more thrust can be generated compared to the previously analyzed model as the top suction surface and bottom pushing surface work together to produce forward force. As described in Section 3.2, lower PR resulted in disappearance of the LEV^D^_L_ and earlier LEV^D^_T_ formation. Lowering the PR leads to a vortex formation pattern that can produce more thrust as the LEV^D^_T_ induced top surface suction becomes stronger and LEV^D^_L_ induced lower surface suction becomes weaker. This helps to explain the difference between thrust production for the baseline case and case 2 seen in Figure 6a and Table 2. 

With an understanding of how reducing PR can lead to significant thrust performance enhancements, the next section aims to combine the simultaneous variation of reducing PR for benefiting thrust and reducing BR to benefit power consumption to further enhance performance.

### 3.4. Hydrodynamic Performance and Surface Forces of Simultaneous Bending and Pitching Variation Case

In this section, the hydrodynamic performance for a model with BR = 0.83, PR = 0.67 will be examined. The BR was selected due to the trough in C_PW_ consumption for the model with BR = 0.83 PR = 1.0 as discussed in Section 3.1. The PR was selected for the observed thrust enhancements due to the timing of LEV^D^_L_ and LEV^D^_T_ formation as discussed in the previous sections. 

**Figure 12:**
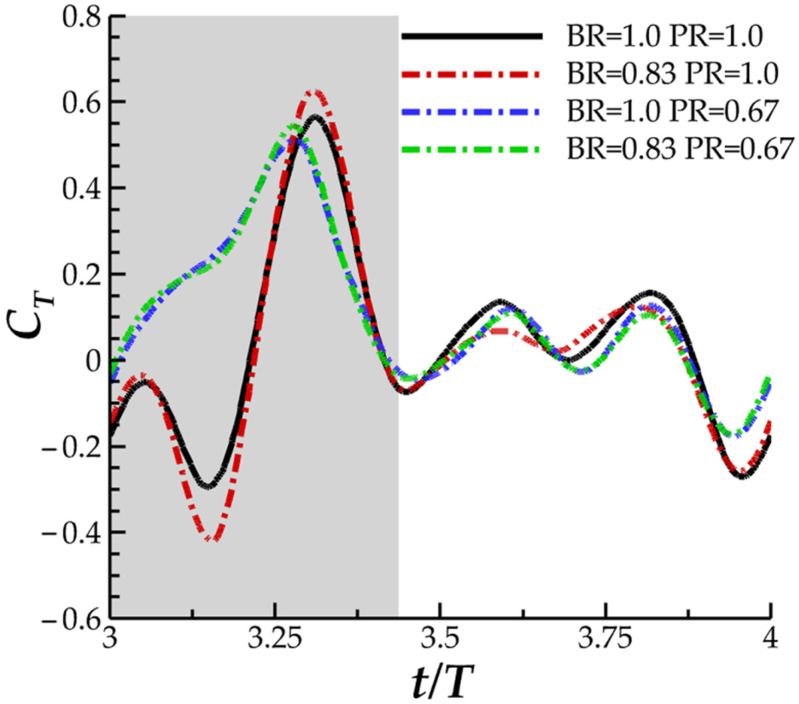
Instantaneous thrust performance comparison for newly added case 7 with BR = 0.83 PR = 0.67.

The force history comparison for four cases is show in in Figure 12: case 7 (BR = 0.83 PR = 0.67), case 2 (BR = 1.0 PR = 0.67), case 4 (BR = 0.83 PR = 1.0), and finally the baseline case for comparison. This way, comparisons between the unscaled BR and PR case, individually scaled BR and PR case, and simultaneously scaled BR and PR case can be made. The newly added case 7 produces thrust that matches the instantaneous thrust production for case 2 almost exactly, as shown in Figure 12. The ‘double-peak’ downstroke thrust feature exhibited for BR = 0.83 in case 4 is not present for case 7, despite case 7 having the same BR but PR scaled to 0.67. Thus, case 7 is expected to provide significant performance benefits compared to case 4. In Table 3, cycle averaged thrust and power consumption values can be found. Entries are normalized by the cycles averaged values for the case with unscaled BR and PR (as in Section 3.1). 

In Table 3, a slight decrease in thrust performance for case 7 is observed compared to case 2. Power consumption is also reduced relative to both cases 2 and 7. This leads to a model with a normalized η  = 2.51, which is 6.3% higher than the most efficient case achieved by varying just PR. 

**Figure 13:**
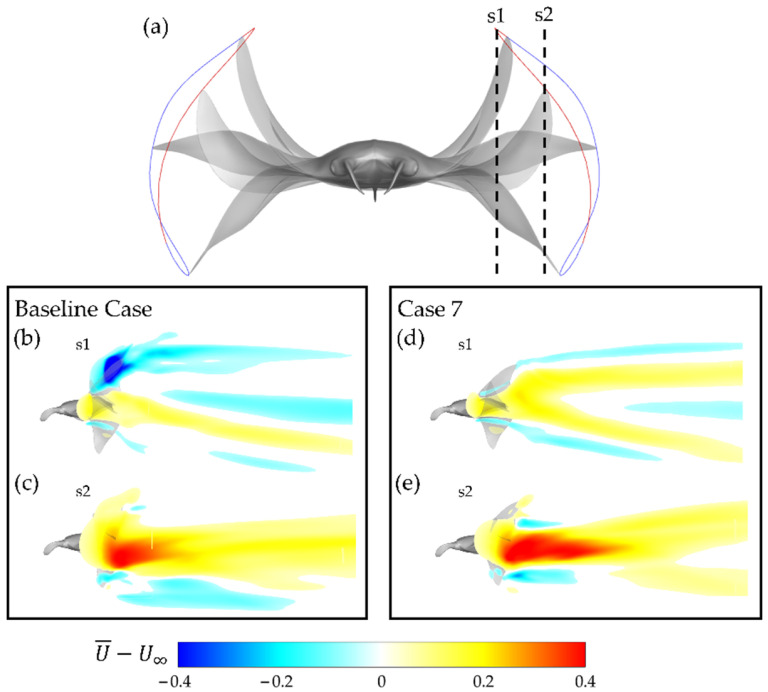
(**a**) Illustration of location of slice cuts relative to the manta body with downstroke motion (red trajectory) and upstroke motion (blue trajectory) visualized; (**b**,**c**) baseline case (BR = 1.0 PR = 1.0) and (**d**,**e**) case 7 (BR = 0.83 PR = 0.67) mean flow at slice cuts.

The mean flow produced by the baseline case and case 7 is compared in Figure 13b,e. Figure 13a illustrates the slice cuts at which the mean flow plots are visualized. S1 represents the velocity field closer to the body and s2 represents the velocity field closer to the tip region of the fin. 

Significant differences are apparent. Firstly, at s1, the baseline case mean flow has a large region in which the mean flow is actually directed upstream. In contrast, case 7 demonstrates primarily downstream-wise mean flow. This indicates that energy is wasted by accelerating fluid opposite the direction of thrust for the baseline case, whereas no such deficiency is present for case 7. At s2 in Figure 13c,e, the mean flows for the baseline case and case 7 are seen to adopt similar geometry. Regions of high mean flow can be observed coincident with approximately the mid up/downstroke. Differences can be seen in the magnitude of the regions of high mean flow, visualized in deep red. The baseline case has a relatively weaker region of faster accelerated flow in comparison to case 7. This supports the previously discussed finding that case 7 has significantly higher thrust production with lower power consumption. 

## 4. Conclusions

In the present study, the hydrodynamic performance and vortex topology of manta ray-like models is investigated through direct numerical simulation. Model manta rays are prescribed motion through pectoral fin skeletal joint rotations. To produce the cases in the study, the manta model pectoral fin kinematics are varied by scaling the pitching ratio (PR) and bending ratio (BR). Significant differences between C_T_ and η are observed for independently varied BR and PR. By manipulating the pitch of the propulsive pectoral fin, cycle averaged thrust production can be maximized for moderately low PR = 0.67 with only a marginal increase in power consumption. This is due to a more even distribution of forward thrust for lower PR while a higher PR leads to thrust production concentrated in the end of the downstroke. The downstroke of the flapping motion is also observed to be a key contributor to the thrust performance of the cases. BR has a less significant impact on thrust. However, power consumption can be minimized at BR = 0.83. Through detailed vortex analysis, the mechanism by which lower PR can improve performance is optimized LEV^D^_L_ and LEV^D^_T_ formation and shedding. Detrimental reverse thrust produced LEV^D^_L_ induced lower surface suction in the early downstroke disappears, while LEV^D^_T_ induced top surface suction strengthens earlier in the downstroke due to earlier LEV^D^_T_ formation. BR = 0.83 PR = 1.0, and BR = 1.0 PR = 0.67 were observed to have benefits individually. Thus, a model utilizing a combination of these scaling factors was subsequently constructed. This model preserves the thrust enhancement of the BR = 1.0, PR = 0.67 model and the power consumption reduction seen in the BR = 0.83, PR = 1.0 model, thereby producing more efficient flapping kinematics. Mean flow plots also demonstrate how case 7 with simultaneous kinematics scaling can more consistently accelerate flow in the downstream direction. 

These findings have implications for a more complete understanding of the propulsive mechanics of the manta ray and for the bio-inspired design of underwater vehicles. The modulation of vortex attachment to pectoral fins through the scaling of pectoral fin kinematics can produce better performance for manta-like robots. More efficient cruising or better burst-thrust performance can be achieved by altering the pectoral fin pitching and bending angles. 

## Figures and Tables

**Table 1:** Tabular description of cases.

Baseline	Fixed BR, Vary PR			Fixed PR, Vary BR			Case 7
	Case 1	Case 2	Case 3	Case 4	Case 5	Case 6	
BR = 1.0	BR = 1.0	BR = 1.0	BR = 1.0	BR = 0.83	BR = 67	BR = 0.5	BR = 0.83
PR = 1.0	PR = 0.83	PR = 0.67	PR = 0.50	PR = 1.0	PR = 1.0	PR = 1.0	PR = 0.67

**Table 2:** Normalized cycle averaged coefficients.

BR and PR		Case 1		Case 2		Case 3		Case 4		Case 5		Case 6	
Normalized $\bar{C_{T}}$		2.27		2.71		2.34		0.65		0.14		−0.53	
Upstroke	Downstroke	0.90	0.10	0.97	0.03	1.11	−0.11	1.12	−0.12	3.04	−2.04	0.31	0.69
Normalized $\bar{C_{PW}}$		1.04		1.14		1.32		0.96		1.02		1.29	
Normalized η		2.19		2.36		1.76		0.68		0.13		--	

**Table 3:** Normalized cycle averaged coefficients.

BR and PR	Case 2	Case 4	Case 7
Normalized $\bar{C_{T}}$	2.71	0.65	2.66
Normalized $\bar{C_{PW}}$	1.14	0.96	0.93
Normalized η	2.36	0.68	2.51

## Data Availability

Not applicable.
